# Transcriptomic analysis of milk somatic cells in mastitis resistant and susceptible sheep upon challenge with *Staphylococcus epidermidis *and *Staphylococcus aureus*

**DOI:** 10.1186/1471-2164-12-208

**Published:** 2011-04-28

**Authors:** Cécile MD Bonnefont, Mehdi Toufeer, Cécile Caubet, Eliane Foulon, Christian Tasca, Marie-Rose Aurel, Dominique Bergonier, Séverine Boullier, Christèle Robert-Granié, Gilles Foucras, Rachel Rupp

**Affiliations:** 1 INRA, UR631, SAGA, F-31326 Castanet-Tolosan, France; 2 INRA, UMR1225, IHAP, F-31076 Toulouse, France; 3 Université de Toulouse; INP, ENVT, UMR1225, IHAP; F-31076 Toulouse, France; 4 INRA, UE321, Domaine expérimental de la Fage, F-12250, Roquefort, France

## Abstract

**Background:**

The existence of a genetic basis for host responses to bacterial intramammary infections has been widely documented, but the underlying mechanisms and the genes are still largely unknown. Previously, two divergent lines of sheep selected for high/low milk somatic cell scores have been shown to be respectively susceptible and resistant to intramammary infections by *Staphylococcus spp*. Transcriptional profiling with an 15K ovine-specific microarray of the milk somatic cells of susceptible and resistant sheep infected successively by *S. epidermidis *and *S. aureus *was performed in order to enhance our understanding of the molecular and cellular events associated with mastitis resistance.

**Results:**

The bacteriological titre was lower in the resistant than in the susceptible animals in the 48 hours following inoculation, although milk somatic cell concentration was similar. Gene expression was analysed in milk somatic cells, mainly represented by neutrophils, collected 12 hours post-challenge. A high number of differentially expressed genes between the two challenges indicated that more T cells are recruited upon inoculation by *S. aureus *than *S. epidermidis*. A total of 52 genes were significantly differentially expressed between the resistant and susceptible animals. Further Gene Ontology analysis indicated that differentially expressed genes were associated with immune and inflammatory responses, leukocyte adhesion, cell migration, and signal transduction. Close biological relationships could be established between most genes using gene network analysis. Furthermore, gene expression suggests that the cell turn-over, as a consequence of apoptosis/granulopoiesis, may be enhanced in the resistant line when compared to the susceptible line.

**Conclusions:**

Gene profiling in resistant and susceptible lines has provided good candidates for mapping the biological pathways and genes underlying genetically determined resistance and susceptibility towards *Staphylococcus *infections, and opens new fields for further investigation.

## Background

Mastitis is defined as an inflammation of the udder, mainly caused by an infection by various bacterial species. Amongst infectious diseases, intramammary infections (IMI) are of major importance in dairy ruminants because of their high frequency and the increased production costs that they incur (loss of milk, treatment, culling). The most prevalent etiological group causing mastitis in sheep is *Staphylococcus *with 78.9% of positive cultures [1]. Coagulase-Negative *Staphylococci *(CNS) are particularly frequent and represent 74.8% of all isolates, most of which are *S. epidermidis *(*Se*) [2]. CNS are considered to be minor pathogens, causing moderate inflammatory responses and often subclinical infections in dairy ruminants [1]. On the contrary, Coagulase-Positive *Staphylococci *(CPS), largely represented by *S. aureus *(*Sa*), are major pathogens in all dairy species. They are principally associated with both chronic and clinical forms of mastitis [3,4], some of which can be very severe and in the worst case lead to a high mortality rate.

Although much work has been carried out in dairy ruminants to understand the complex physiological and cellular events that occur in the mammary gland in response to pathogens [3-5], the protective mechanisms are still obscure. Schematically, when pathogens enter the udder lumen via the teat canal, they are detected by both immune and non-immune cells, and this is followed by the release of chemoattractants. As a consequence, neutrophils migrate from the blood flow to the infection site [4]. These cells can phagocyte bacteria and exert bactericidal activities by releasing potent oxidative products [6]. This massive recruitment of neutrophils in the udder incurs a dramatic increase in the milk somatic cell count (SCC) [5]. Accordingly, SCC has been widely advocated as an easy-to-measure tool for predicting mastitis and discriminating between chronically infected and non-infected animals [5,7]. Recent advances in microarray technology, that nowadays enable the expression analysis of thousands of infection-related genes, have provided novel insights into host response to pathogens. Microarray analysis is a well-adapted technology to investigate the gene regulation mechanisms underlying immunity against pathogens. Previously, gene expression profiles for challenged mammary tissue [8-11], milk cells [12] and peripheral blood mononuclear cells [13] have been studied using microarrays.

There is overwhelming evidence that the host's response to IMI is under genetic control, as extensively described in earlier studies [14-18]. Genetic parameters have been established for milk somatic cell scores (SCS) and occurrence of clinical mastitis, thus indicating that five to twenty percent of all variability between individuals is of genetic origin. Additionally, numerous quantitative trait loci (QTL) for udder health traits have been identified [16,19]. However, up to date, only one of these QTL - the forebrain embryonic zinc finger-like gene -has been fully characterised [20]. Apart from this QTL, the genetic basis of resistance is still largely unknown.

Nevertheless, breeding programmes for mastitis resistance have been implemented throughout the world in dairy cattle [14,16] and sheep [21] using indirect predictor traits such as clinical mastitis and SCS. To assess the effect of SCS-based selection for resistance or susceptibility to IMI, two divergent lines of dairy sheep were created on the basis of their parents' breeding values for SCS [22]. Evaluation of the frequency and duration of mastitis in the two lines demonstrated that selection for decreased SCS is associated with a decrease of IMI [22].

In the present study, we performed transcriptomic analysis of milk somatic cells (MSC), collected from mastitis resistant and susceptible ewes using a generic 15K oligonucleotide chip. MSC were collected after challenge with *Se *and *Sa *during the first and second lactations respectively. Our objective was to use this animal-model of divergent-SCS-lines in order to identify some of the genes and molecular mechanisms involved in the genetic basis of the protective host response to staphylococcal IMI.

## Results

### Experimental challenges

Clinical examination of the mammary gland, bacteriological analyses and SCC confirmed that all animals were free from udder infections before challenges.

**Figure 1:**
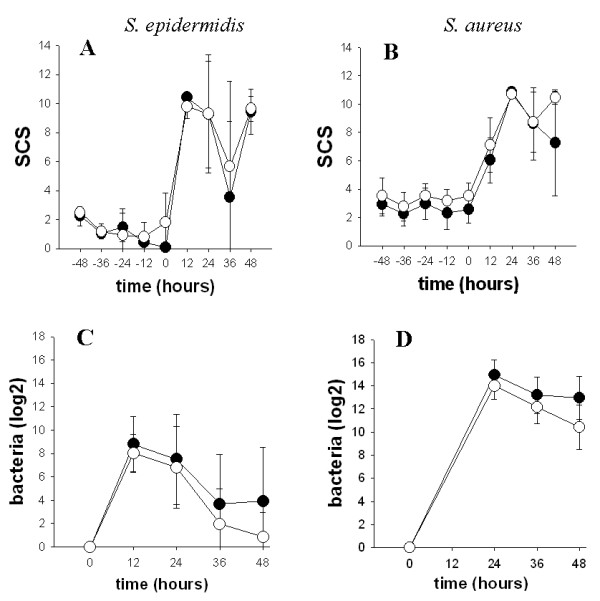
**Evolution of SCS and bacteriology titres before and after *S. epidermidis *and *S. aureus *challenges**. A and B. SCC were measured in the inoculated half-udder from 48 hours before to 48 hours after challenge. SCS were computed from the SCC with a log-2 transformation and their time evolution are drafted on the graph. C and D. Bacteriology counts were measured at the same time points (the time 12 hours post-inoculation with *S. aureus *is missing). All mammary glands were free of infection before inoculation. The positive values were transformed in score by a log-10 formula. The resistant line is represented in open symbols and the susceptible line in closed symbols. Figures A and C correspond to *S. epidermidis *and Figures B and D correspond to *S. aureus *challenge.

At the beginning of the first infection with *Se*, the SCS increased rapidly in the inoculated half udder (Figure 1A). It then increased at a slower rate after the second infection with *Sa *in the contra-lateral half udder (Figure 1B). Consequently, the SCS were higher upon *Se *than upon S*a *challenge at 12 hours (μ_Se _= 10.1 ± 0.7, μ_Sa _= 6.6 ± 1.0, p < 0.001), although the contrary was observed at 24 hours (μ_Se _= 9.3 ± 3.7, μ_Sa _= 10.8 ± 0.19, p = 0.18). Bacteriological titres (Figure 1C and 1D) were significantly higher after *Sa *challenge than after *Se *challenge over the period from 24 to 48 hours (μ_sa _= 12.9 ± 2.0, μ_se _= 4.1 ± 4.1, p < 0.001).

No significant differences were observed for milk SCS between the resistant and susceptible lines, either before or after the two successive challenges.

By contrast, the bacteriological titres were significantly higher in the susceptible line than in the resistant line over the period from 24 to 48 hours upon S*a *challenge (μ_susceptible _= 13.7 ± 1.7, μ_resistant _= 12.2 ± 2.1, p = 0.048). Although it was not significant, the bacteriological titre was also higher at 48 hours upon S*e *challenge in the susceptible line when compared to the resistant line (μ_susceptible _= 3.9 ± 4.6, μ_resistant _= 0.86 ± 2.1, p = 0.27). These results suggest that bacterial clearance is more efficient in the resistant than in the susceptible line.

**Figure 2:**
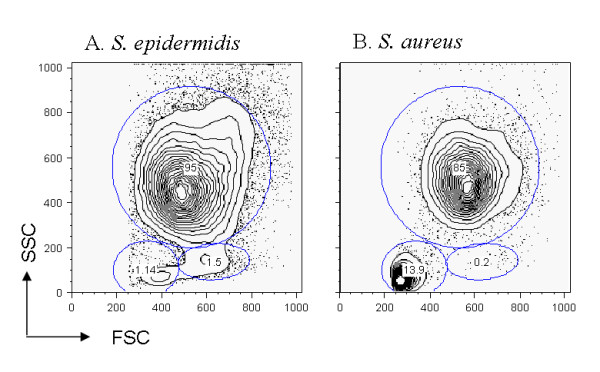
**Cell population in the milk after *S. epidermidis *and *S. aureus *challenges**. After incubation with propidium iodide, cells from cisternal lavages were analysed by flow cytometry. Dead cells were electronically gated out, and cell types (granulocytes, monocytes/macrophages and lymphocytes) were analysed on the forward and side scatter intensity profiles. The results from a resistant ewe after *Se *(A) or *Sa *(B) are presented.

Furthermore, a difference in cell viability between challenges was observed in the milk cells collected by cisternal lavage 12 hours after challenge, with respectively 92.1% ± 6.1 and 55% ± 17.1 of viable cells in *Se *and *Sa *challenges (p < 0.001). Notably, the proportion of lymphocytes was higher after *Sa *than after *Se *infection (7.8% ± 4.9 and 4.1% ± 11, respectively, p < 0.001) as illustrated in Figure 2 for one representative ewe. Despite the differences observed between the challenges, the profile of cell types was not significantly different between the two divergent lines, except for monocytes/macrophages whose proportion tended to be higher in susceptible animals (p = 0.28).

### Comparison of the host response to *S. aureus *and *S. epidermidis *challenges

#### Microarray analysis and biological interpretation

**Figure 3:**
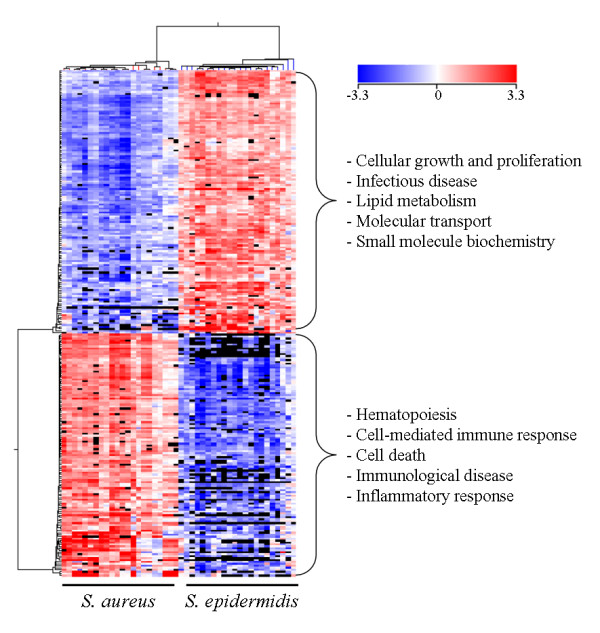
**Heatmap of differentially expressed probes in samples from *S. epidermidis *and *S. aureus *challenges**. Hierarchical clustering was performed using Pearson-centred unsupervised statistics with GeneSpring^®^. Gene expression intensities are in rows (n = 261, FDR q-value < 0.05 and aFC > 5). Each column represents a sample. The level of gene expression is proportional to the colour scale. The genes in the top part of the graph are over-expressed in *Se *when compared to *Sa *samples, whereas it is the contrary for the bottom part of the graph. The cluster tree of the genes (left) illustrates the nodes of genes co-regulated in each *Staphylococcus *infection and their main functions are indicated. The cluster tree of samples above the heatmap graph enabled a perfect discrimination between *S. aureus *and *S. epidermidis-*challenged samples.

The functional roles of MSC in response to different *Staphylococcus spp *were explored through gene expression profiling in the divergent sheep lines successively infected with *Se *during the first lactation, and *Sa *during the second lactation. The multifactorial ANOVA applied probe by probe identified 5,573 probes as differentially expressed according to the challenge effect (FDR of 5% and absolute fold-change (aFC) > 1.5; the FC is the ratio between *Sa *and *Se *challenges). Among these, 261 probes had an aFC > 5 (Additional file 1). The DE probe list enabled a perfect segregation between animals challenged by *Se *and *S*a as shown by the hierarchical clustering in Figure 3. This probe list corresponded to 210 annotated genes with 95 and 115 genes that were more expressed in *Sa *and *Se *infections, respectively. The main functions of the genes expressed at a higher level in *Sa *than in *Se *infections were associated with the immune response: hematopoiesis (p = 0.010), cell-mediated immune response (p = 0.009), cell death (p = 0.001), immunological disease (p = 0.001) and inflammatory response (p = 0.010). Genes whose expression was higher after *Se *challenge were linked to cellular growth and proliferation (p = 0.048), infectious disease (p = 0.032), lipid metabolism (p = 0.042), molecular transport (p = 0.049) and small molecule biochemistry (p = 0.048) (Ingenuity Pathway Analysis - IPA - data, not shown).

Network analysis with IPA produced two networks. In the first network (Additional file 2A), which is characterised by cell-to-cell signalling and interaction and cell-mediated immune response, the T cell receptor signalling pathway (with the genes: *cd247, cd3d, cd3e, cd3g, ctla4, itk, ppp3cc, rasgrp1, tra@, zap70*) and the major histocompatibility complex - MHC - pathway (with six sub-units: *dma, dqa1 *(three probes), *dqa2 *(two probes), *dra*, *drb1 *(two probes) and *drb3*) are highly represented. In a second network, defined by inflammatory disease and response and haematological disease, both the IL1 receptor pathway and the TNF pathway are well represented with *il1r1, il1rap *and *irak4*, and *traf2-3-5 *respectively (Additional file 2B).

#### Real-time qPCR validation of the differentially-expressed genes between *Sa *and *Se *challenges

Real-time qPCR was used to confirm the gene expression differences between the two challenges. The most expressed gene in *Se *challenges was *cpb2*. We also examined the differential expression of chemokine (C-C motif) receptor 3 (*ccr3*) and interleukine 1 receptor type II (*il-1r2*) for their role in pathogen detection, as well as myxovirus resistance 2 (*mx2*) and granzyme H (*gzmh*) for their role in immune response. Hence, five genes were selected, three of which were more expressed in the *Sa *condition (*ccr3*, *gzmh *and *mx2*) and two in the *Se *condition (*cpb2 *and *il-1r2*). Chemokine (C-X-C motif) ligand 10 (*cxcl10*) was added to this gene list since it is known to induce T cell recruitment in inflamed tissue, and because it had been discarded from the microarray analysis due to missing data (data not shown). The differential expression between the two challenges was significantly (p < 0.05) confirmed for five out of the six genes (Table 1). Among those genes, the expression of *cxcl10 *was 60-fold higher in *Sa *than in *Se *challenges. Although not significant (p = 0.12), the expression of *gzmh *was higher in the *Sa *than the *Se *condition (Table 1).

**Table 1:** RT-qPCR results for the differentially expressed genes between *S. aureus *and *S. epidermidis *challenges

Gene	*S. aureus*	*S. epidermidis*
**CXCL10**	91.46 ± 70.08***	1.46 ± 1.79
**IL1R2**	0.12 ± 0.11***	2.09 ± 2.35
**CPB2**	0.05 ± 0.04***	1.40 ± 1.84
**CCR3**	6.69 ± 6.80**	1.35 ± 0.83
**MX2**	4.64 ± 4.75**	1.00 ± 1.07
**GZMH**	12.41 ± 12.12	2.98 ± 2.52

The results represent the mean ± standard deviation of the relative expression in qPCR of six differentially expressed genes identified in the microarray analyses between *Se *and *Sa *challenges. A non parametric Wilcoxon test was performed with SAS to identify the differentially expressed genes.

*** p-value < 0.01; ** p-value < 0.05.

### Comparison of gene expression profiles between mastitis resistant and susceptible lines

#### Microarray analysis

Using a multifactorial ANOVA model applied probe by probe, 57 probes were shown to be significantly DE between the resistant and susceptible lines (FDR 5% and aFC > 1.5) (Table 2). These probes corresponded to 52 annotated genes, with 33 and 19 genes expressed at a higher level in the resistant and susceptible line, respectively. This list of 52 genes is further named as the "main list". Out of the 33 genes with higher expression in the resistant line, eight had a FC ≥ 2 (*cryl1, tp53, a non-classical mhc*-I, *slc40a1, eif4ebp1, ppapdc1b, slc46a3 *and *loc784517*). Only three out of the 19 genes that were expressed at a higher level in the susceptible line had an aFC > 2 (*gtpbp4, mapre1, tmem87b*).

**Table 2:** List of the differentially expressed genes between the resistant and susceptible lines

ProbeName	Genbank	Genes	**Fold change**^**1**^	FDR	Description
A_70_P018246	FE029767	CRYL1	4.7	0.030	crystallin, lambda 1
A_70_P062021	EE803126	TP53	4.6	0.031	tumor protein p53
A_70_P001626	CN824748	BOLA-NC1	3.5	0.027	non-classical MHC class I antigen
A_70_P007316	EE851499	SLC40A1	2.6	0.046	solute carrier family 40 member 1-like iron-regulated transporter
A_70_P029426	CN822074	EIF4EBP1	2.4	0.014	eukaryotic translation initiation factor 4E binding protein 1
A_70_P049136	DY522411	KIAA2013	2.2	0.024	
A_70_P054531	EE849541	PPAPDC1B	2.2	0.000	phosphatidic acid phosphatase type 2 domain containing 1B
A_70_P013986	EE808866	SLC46A3	2.1	0.040	solute carrier family 46, member 3
A_70_P010846	EE822719		2.0	0.047	
A_70_P006576		LOC784517	2.0	0.030	similar to cationic amino acid transporter 5;
A_70_P059451	EE748438	RARΑ	2.0	0.027	retinoic acid receptor, alpha
A_70_P066641	FE023374	CCDC125	1.9	0.019	coiled-coil domain containing 125
A_70_P019936	EE776127	KDM4B	1.8	0.023	lysine (K)-specific demethylase 4B
A_70_P038196	EE746703	SULT1A1	1.8	0.041	sulfotransferase family, cytosolic, 1A, phenol-preferring, member 1
A_70_P031756	EE767595	YPEL3	1.8	0.040	yippee-like 3
A_70_P062891	EE747727	PIGR	1.8	0.027	polymeric immunoglobulin receptor
A_70_P021746	EE782837	ACTN4	1.8	0.040	actinin, alpha 4
A_70_P055391	DY492111	FAM100B	1.8	0.023	Family with sequence similarity 100, member B
A_70_P021086	EE826005	PCID2	1.7	0.048	PCI domain containing 2
A_70_P069621	EE856030		1.7	0.049	
A_70_P054671	EE765024	LOC781337	1.7	0.019	
A_70_P038536	EE827115	GABARAPL1	1.7	0.026	GABA(A) receptor-associated protein like 1
A_70_P022126	EE826386	RERE	1.7	0.040	similar to atrophin-1 like protein
A_70_P059286	DY500392	PLOD1	1.6	0.027	procollagen-lysine 1, 2-oxoglutarate 5-dioxygenase 1
A_70_P064491	EE836176	SERINC3	1.6	0.040	serine incorporator 3
A_70_P024181	DQ239612	TUBA1A	1.6	0.045	tubulin, alpha 1a
A_70_P011346	EE790238	STAB1	1.6	0.041	stabilin 1
A_70_P016501	EE873028	PTTG1IP	1.6	0.030	pituitary tumor-transforming 1 interacting protein
A_70_P060561			1.6	0.030	
A_70_P042031	GO760287	VAMP5	1.6	0.041	vesicle-associated membrane protein 5
A_70_P045551	EE792489	LOC507126	1.6	0.042	basement membrane-induced gene
A_70_P033276	EE812467	PPIL3	1.6	0.019	peptidylprolyl isomerase cyclophilin-like 3
A_70_P007306	EE843558		1.6	0.030	
A_70_P019896	EE823241	UQCRQ	1.6	0.025	low molecular mass ubiquinone-binding protein (9.5 kD)
A_70_P023216	EE864116	CNNM2	1.6	0.040	cyclin M2
A_70_P066801	EE865060	DEF8	1.6	0.030	differentially expressed in FDCP 8 homolog
A_70_P049271	EE806359	GIYD1	1.5	0.030	GIY-YIG domain containing
A_70_P060881	EE824343	ZNF259	-1.5	0.040	zinc finger protein 259
A_70_P060761	EE749912	ARMC1	-1.5	0.019	armadillo repeat containing 1
A_70_P064541	EE833852	PPIG	-1.6	0.049	peptidylprolyl isomerase G cyclophilin G
A_70_P063461	FE022716	NVL	-1.6	0.040	nuclear VCP-like
A_70_P062791	EE823755	POLR2D	-1.6	0.025	polymerase (RNA) II (DNA directed) polypeptide D
A_70_P050356	EE777866	CYP51A1	-1.6	0.025	cytochrome P450, family 51, subfamily A, polypeptide 1
A_70_P009601	EE747016	STT3A	-1.6	0.040	STT3, subunit of the oligosaccharyltransferase complex, homolog A
A_70_P050201	FE030100	DNTTIP2	-1.7	0.040	deoxynucleotidyltransferase, terminal, interacting protein 2
A_70_P046246	EE777707	TIMM8A	-1.7	0.026	translocase of inner mitochondrial membrane 8 homolog A
A_70_P061706	EE815731	USP10	-1.7	0.045	ubiquitin specific peptidase 10
A_70_P057996	EE827511	FYN	-1.7	0.041	FYN oncogene related to SRC
A_70_P060371	EE849850	AHCYL1	-1.7	0.000	adenosylhomocysteinase-like 1
A_70_P049176	EE746595	HMGCS1	-1.7	0.013	3-hydroxy-3-methylglutaryl-Coenzyme A synthase 1 (soluble)
A_70_P049891	CF118151	ITGA2	-1.8	0.040	integrin, alpha 2
A_70_P034661	EE780305	HOOK1	-1.9	0.030	hook homolog 1 (Drosophila)
A_70_P011861	FE031423	MUC12	-1.9	0.033	mucin 12, cell surface associated
A_70_P055431	EE849843	FYN	-1.9	0.040	FYN oncogene related to SRC
A_70_P010631	EE756345	GTPBP4	-2.1	0.019	GTP binding protein 4
A_70_P006201	FE031048	MAPRE1	-3.1	0.014	microtubule-associated protein, RP/EB family, member 1
A_70_P057056	EE780570	TMEM87B	-3.5	0.030	transmembrane protein 87B

^1 ^In the fold-change, the enumerator is the resistant line and the denominator is the susceptible line.

ANOVA models with Line and Challenge effects were applied probe by probe with GeneSpring^® ^(n = 57 probes, n = 52 genes, FDR q-value < 0.05, absolute FC^1 ^> 1.5).

**Figure 4:**
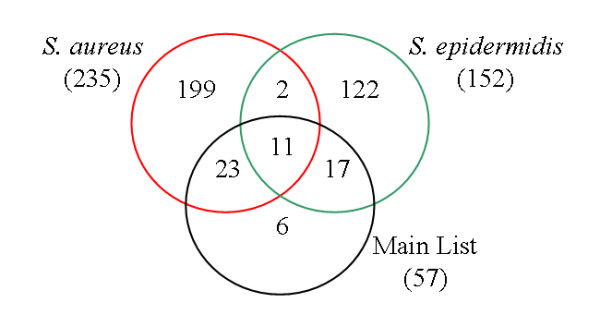
**Venn diagram of the differentially expressed genes between the resistant and susceptible lines**. The three lists of differentially-expressed genes between the lines were compared using a Venn diagram: the main list (n = 57 probes, FDR q-value < 0.05, aFC > 1.5), and the list from the single-challenge analysis *S. aureus *and *S. epidermidis *(t-test, p < 0.01, aFC > 1.5, n_Sa _= 235 probes and n_Se _= 152 probes, respectively). A total of 380 probes are represented.

As the gene expression of MSC response differs between challenges with two different *Staphylococcus *species, the line effect was also analysed independently within each challenge. The lesser amount of data in separate analyses made it necessary to loosen the significance threshold to find differentially expressed genes within *Se *or *Sa *challenges. Accordingly, a total of 152 probes (138 annotated genes) was considered to be DE between the lines after *Se *challenge and 235 probes (204 annotated) after *Sa *challenge (p-value ≤ 0.01 and aFC > 1.5) (Additional file 3). The latter two lists were compared to each other and also to the main list (57 probes) by generating a Venn diagram (Figure 4). Nine genes (*bola-nc1, ccdc125, eif4ebp1, kdm4b, mapre1, ppapdc1b, ppil3, timm8a, tmem87*) and also *loc784517 *and an unannotated probe were common to the three lists. Forty other genes belonged to the Main List and one of the two single-challenge lists (Figure 4).

To gain further insight into the biological signification of the differences between the resistant and susceptible lines, information from the separate analyses in *S*a an *Se *was added to the main list. The three probe lists were pooled together (Additional file3), resulting in a super-list of 335 annotated genes (380 probes). In this list, further named as the "pooled list", 209 and 169 genes were expressed at a higher level in the resistant and the susceptible animals, respectively.

#### Real-time qPCR validation of the differentially-expressed genes between resistant and susceptible lines

To confirm the line effect, seventeen genes were analysed by qPCR. Genes were chosen because they belonged to the main list *(cryl1, eif4ebp1, gtpbp4, mapre1, ppapdc1b, rarα, slc40a1, tmem87b *and *tp53)*, or had been identified in *Sa *(*ccl5, itgb6, s100a2, saa2 *and *tlr2*) or *Se *conditions (*capn3, psmd4 *and *st3gal4*) (Table 3). Real-time qPCR confirmed the significant (p < 0.05) differential expression of eight genes (*capn3*, *cryl1*, *itgb6, psmd4, rarα*, *saa2, st3gal4 *and *tp53*). Six others (*ccl5, gtpbp4, ppapdc1b, s100a2, slc40a1 *and *tmem87b) *were close to signification (p < 0.10). The genes *tlr2, eif4ebp1 *and *mapre1 *were not significantly confirmed by qPCR (0.20 < p < 0.30), however the relative gene expression between resistant and susceptible was in accordance with microarray analysis (Table 3).

**Table 3:** RT-qPCR of the differentially expressed genes between resistant and susceptible lines

Microarray result	Genes	All data		*Sa *data		*Se *data	
		Resistant	Susceptible	Resistant	Susceptible	Resistant	Susceptible
	CRYL1	26.50 ± 29.98**	4.65 ± 5.68	40.39 ± 37.92**	6.20 ± 6.66	12.61 ± 8.71*	3.10 ± 4.57
	TP53			4.53 ± 3.51**	1.16 ± 0.72		
	RARα	1.34 ± 0.82**	0.76 ± 0.37	0.95 ± 0.58*	0.51 ± 0.31	1.72 ± 0.90*	1.02 ± 0.20
	SLC40A1			5.07 ± 8.07*	1.29 ± 1.10		
**Main List**	GTPBP4			0.59 ± 0.47*	1.21 ± 0.92		
	TMEM87B			0.73 ± 0.14*	1.19 ± 0.86		
	PPAPDC1B	0.43 ± 0.39	0.81 ± 0.92	0.25 ± 0.13	0.26 ± 0.19	0.61 ± 0.50*	1.36 ± 1.05
	EIF4EBP1	1.54 ± 0.80	1.27 ± 0.75	1.93 ± 0.75	1.49 ± 1.01	1.14 ± 0.67	1.05 ± 0.33
	MAPRE1			0.87 ± 0.57	1.16 ± 0.76		
	SAA2			0.33 ± 0.31**	1.86 ± 2.30		
	ITGB6			0.42 ± 0.31**	1.49 ± 1.49		
***Sa *list**	S100A2			0.58 ± 0.39*	1.28 ± 0.94		
	CCL5			0.55 ± 0.36*	1.35 ± 1.02		
	TLR2			2.04 ± 2.17	1.09 ± 0.47		
	CAPN3	3.63 ± 2.71**	1.52 ± 0.98	4.07 ± 3.23	1.66 ± 0.81	3.19 ± 2.28*	1.38 ± 1.18
***Se *list**	PSMD4	0.82 ± 0.28**	1.06 ± 0.31	0.96 ± 0.27	1.08 ± 0.33	0.67 ± 0.22**	1.04 ± 0.32
	ST3GAL4	1.56 ± 1.31**	0.82 ± 0.50	0.96 ± 0.73	0.54 ± 0.37	2.16 ± 1.55**	1.10 ± 0.48

The results represent the mean ± standard deviation of the relative expression in qPCR of seventeen differentially expressed genes identified in the microarray analyses with all data (main list), *S. aureus *(*Sa *list) or *S. epidermidis *data (*Se *list). qPCR were performed with *Sa *or *Se *samples or with both *Sa *an *Se *samples (All data). A non parametric Wilcoxon test was performed with SAS to identify the differentially expressed genes. ** p-value < 0.05; * p-value < 0.10.

#### Clustering of the differentially expressed genes between the resistant and susceptible lines

**Figure 5:**
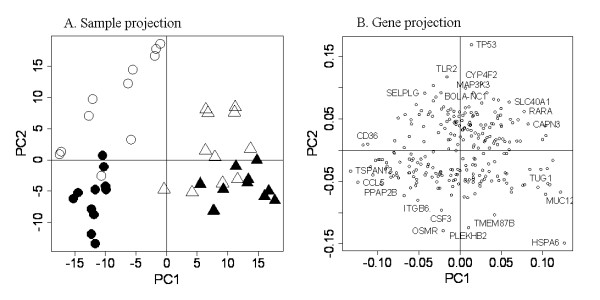
**Principal component analysis of the differentially-expressed probes between resistant and susceptible lines**. PCA was performed with R on the 380 probes that are differentially expressed between the lines from the pooled list. (A) All samples from the four conditions - Low-SCS animals infected by *Se *(open triangle), Low-SCS animals infected by *Sa *(open circle), High SCS animals infected by *Se *(closed triangle) and High SCS animals infected by *Sa *(closed circle) were separated based on Line-Challenge along the principal component 1 (PC1) and PC2 axes. PC1 explained 11.2% of the total variations and mainly discriminates the challenges whereas the PC2 explained 7.8% of the total variations and segregates between the lines. (B) The 380 probes were projected on PC1 and PC2.

Principal component analysis (PCA) of the probes from the pooled list revealed that the first two principal components, that represented 19% of the total variations, could separate the sheep samples into three clusters: resistant animals infected by *Sa*, susceptible animals infected by *Sa *and animals infected by *Se *(Figure 5A). The principal component 1 (PC1) explained 11.2% of the total variations and clearly discriminated the *Staphylococcus spp *within the differentially expressed genes between the lines (Figure 5A). The gene expression of *capn3 *was mainly associated with *Se *challenge whereas the gene expression of *ccl5 *and *cd36 *was linked to *Sa *challenge (Figure 5B). The PC2 explained 7.8% of the total variations and generally tended to separate the resistant and susceptible animals whatever the challenge (Figure 5A). Gene expression of *tp53, tlr2, map3k3, selplg *and *bola-nc1 *was associated with the resistant animals whereas gene expression of *plekhb2, tmem87b *and *csf3 *was linked to the susceptible ones (Figure 5B).

#### GO and pathway analysis of the differentially expressed genes between resistant and susceptible lines

Additional biological information was obtained using the Database for Annotation, Visualization and Integrated Discovery (DAVID v6.7) with human ortholog gene names - HUGO nomenclature (n = 306 out of 335 genes recognized by DAVID). First, Gene Ontology (GO) analysis of the biological process terms was performed. The up-regulated genes in the resistant line showed a significant enrichment of leukocyte adhesion and activation, cell death regulation, intracellular signalling cascades and negative regulation of macromolecule metabolic processes and catalytic activities (Additional file 4). On the contrary, the down-regulated genes showed a significant enrichment in positive regulation of inflammatory response (p = 0.029) (Additional file 4). Both up and down-regulated genes showed an over-representation of genes involved in the regulation of transcription and RNA maturation and in cell motion (Additional file 4).

Two BIOCARTA pathways were well-represented: apoptotic signalling in response to DNA damage (p = 0.029, with *akt1, eif2s1, cycs, tp53*), and adhesion and diapedesis of granulocytes (p = 0.087, with *csf3, selp, selplg*). Four KEGG pathways were also identified: lysosome (p = 0.010, with *tcirg1, sgsh, cd68, smpd1, ctsd, ctsa, ctsb, fuca1*), adipocytokine signalling pathway (p = 0.012, with *akt1, cd36, mapk8, acsl4, acsl3, acsl5*), hematopoietic cell lineage (p = 0.032, with *csf3, cd36, itga5, cd59, itga2, csf1r*) and focal adhesion (p = 0.054, with *akt1, lama3, ccnd3, actn4, itga5, fyn, itgb6, itga2, mapk8*).

#### Gene network analysis of the differentially expressed genes between resistant and susceptible lines

**Figure 6:**
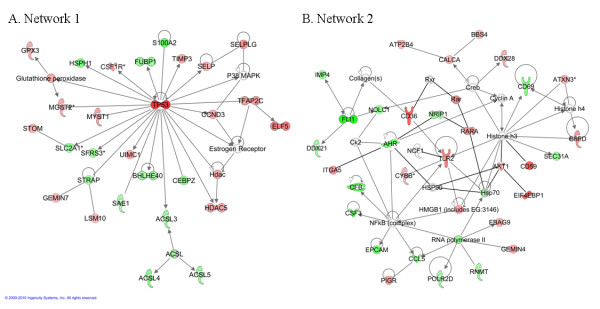
**Network analysis of the differentially-expressed genes between resistant and susceptible lines**. Network analysis was performed with IPA (n = 335 genes, n = 287 IPA network eligible genes). The colours represent the expression level: the genes over-expressed in resistant animals are in red whereas the down-regulated genes are in green. (A) Twenty-eight genes belonged to network A that scores 42. The main biological functions are lipid metabolism, molecular transport and small molecule biochemistry. (B) Thirty-five genes are present in network B. The original network involved twenty-six genes and scores 39. It is characterised by cell movement, haematological system development and function, and immune cell trafficking. We could add seven DE expressed genes of interest (*akt1, cd59, eif4ebp1, hspa6, itga5, osmr *and *rarα*) to this network through five other genes, with direct relationships with genes involved in this network.

Systemic identification and grouping of line-associated genes into biological networks was performed with IPA using HUGO names (n = 331 out of 335 genes, recognized by IPA). Five networks were obtained with a score superior to 30. Twenty-two to twenty-eight genes were involved in each network. The two first networks are presented in the Figure 6. Network 1 is characterised by lipid metabolism, molecular transport and small molecule biochemistry (Figure 6A). It presents *tp53*, the second most up-regulated gene in the resistant line (Table 2 and Additional file 3), as a hub which means that *tp53 *regulates or is regulated by a large proportion of the identified DE genes (Figure 6A). Network 2 is defined by cellular movement, haematological system development and function, and immune cell trafficking (Figure 6B). It highlights a central position for *tlr2*, linked to *cd36*, that are of major importance for pathogen recognition. Other genes were included in this network *eif4ebp1 *and *itga5*, *nfkb *(immune response), *ccl5 *(diapedesis) and *rarα *and *ahr *involved in the retinoic acid pathway (Figure 6B).

#### Communal transcription factor analysis

To enhance the understanding of the genetic basis of the DE genes, the potential transcription factors that can regulate these genes were investigated by looking for transcription factor-binding sites (TFBPS) that were over-represented in the promoter sequences of the DE genes. Using 260 genes recognised in InnateDB, significant enrichment was demonstrated for 11 transcription factors with a p < 0.05 (CEBPB, PAX5, E4F1, CREB1, ETV7, HIF-1, SMAD1, FOXO4, NRF-2, NRF1 and NFIC), and nine other transcription factors with p < 0.10 (AP-1, ETS1, 120-kDa CRE-binding protein, FOXO1A, NKX2-5, ATF, ATF3, XBP1 and PBX1). Furthermore, the transcription factors AHR, which showed lower expression levels in the resistant animals, and CEBPA, which showed higher expression levels, could bind the promoters of thirty-three and seven genes, respectively, that were more expressed in the susceptible line (p = 0.07 and p < 0.1, respectively). TP53, which showed higher expression levels in the resistant animals, could bind fourteen up-regulated genes (p = 0.16).

## Discussion

### Gene expression of milk somatic cells

In the present study, we compared the gene expression in MSC upon mammary challenge. Up to date, only a small number of studies have analysed the transcriptome of MSC, by comparing infected and non-infected glands [16,17]. MSC populations, however, are highly modified after infection: whereas mononuclear cells are preponderant in healthy glands [23], neutrophils represent the main cell type in infected glands [4,13,24]. Because of the profound modifications within the MSC population, the comparison of MSC gene expression before and after challenge should be interpreted with caution as they are the result of mixed molecular and cellular effects. Hence, our study focused on the transcriptomic differences within homogeneous cell populations between the lines upon challenge. It is well known that the development of *S. aureus *in any tissue implies host cell apoptosis [25,26], and so RNA breakdown occurs. To circumvent the difficulty, cisternal lavage was performed after emptying the mammary gland to gather recently emigrated and living MSC. This protocol enabled us to recover a higher proportion of living cells and obtain good quality RNA, despite the short half-life of milk neutrophils.

Physiological mechanisms that are mobilised during the early response are considered as determinant for the outcome of IMI. Therefore, we selected an early time point (12 hours) for cell collection, during the first wave of cell recruitment, just before the bacteria titres diverge, and the side effects, on the mammary tissue due to bacteria growth, appear. In addition at 12 hours post inoculation, the milk cell population was much more homogeneous than at later time points, *i.e. *95% of the cells were neutrophils. Therefore, despite the small number of animals used and the fact that only one time point was considered, we identified lists of significant and biological relevant differentially expressed genes in MSC. The results gave some insight into the differential response of the host to IMI according to the mastitis causing pathogen and its genetic background.

### Differences between *S. epidermidis *and *S. aureus *challenges

Although genetically *Sa *and *Se *are closely related [27], the host cell viability after *Sa *challenge was lower than after *Se *challenge and the proportion of T-cells was higher. This is correlated with the striking differences in the gene expression profiles of infected MSC at twelve hours post-inoculation. Indeed, 5,573 genes were differentially expressed in MSC infected either by *Sa *or *Se. *The greatest absolute fold changes (aFC) were achieved when *Sa *was compared to *Se *- 91% of the genes with an aFC > 10 were over-expressed after *Sa *challenge. A high number of DE genes underlined T cell recruitment after *Sa *challenge. Indeed, two major genes that are up-regulated after *Sa *challenge are involved in T-cell responses: the chemokine (C-X-C motif) ligand 10 (*cxcl10*) [28] and the cytotoxic T lymphocyte-associated protein 4 gene (*ctla4*). The up-regulation of *ctla4 *in MSC of cows with chronic *Sa *mastitis has already been reported [13]. In addition, the components of the chemokine signalling and cell adhesion molecule pathways were over-represented after *Sa *challenge. These pathways play important roles in blood neutrophil arrest and diapedesis across the endothelium [6]. Furthermore, cytokine-cytokine receptor interactions are also noticeable and cytokines are known to tightly regulate neutrophil functions during inflammatory response [6]. Thus, pro-inflammatory cytokines lead to the activation of the mitogen-activated protein kinase pathway (MAPK) in neutrophils and promote leukocyte recruitment to inflammation sites. Furthermore, *S. aureus *exerted an oxidative priming and a pro-apoptotic effect on neutrophils, contrary to *S. epidermidis *strains [29]. *S. aureus *cytotoxicity mainly depends on proteases, hyaluronidases, lipases and nucleases that facilitate tissue destruction, membrane-damaging toxins that cause cytolytic effects in host cells, and superantigens that contribute to the symptoms of septic shock [30]. Moreover, three sub-units of the major histocomptibility complex (MHC) class II (DQA1, DQA2 and DRB1) were up-regulated after *Sa *challenge suggesting recruitment of antigen-presenting cells or activation of T cells. The higher proportion of lymphocytes after *Sa *challenge might be partly responsible for the large variations of gene expression observed between *Sa *and *Se *challenges.

Altogether, the gene expression profiles of MSC upon challenge with two distinct pathogens differed widely in relation with the severity of the mastitis, bacterial counts and milk SCC [7,22]. Furthermore, it should be noted that all the differences of MSC gene expression infected either by *Sa *or *Se *might not be caused by the *Staphylococcus *species. Actually, other factors may explain these differences since the animals were infected twice, and a memory response could have developed consequently to the first challenge. However, this is improbable due to the recurrence of infections frequently reported in apparently susceptible animals and the poor effect of vaccination with living bacteria on the occurrence and severity of subsequent infections. The animals' physiological conditions (age, effect of previous infection, *etc*.) at the second lactation could also influence the pattern of response. So partial confusion with other effects not related to the bacterial strain cannot be totally excluded.

### Transcriptome differences associated with mastitis resistance or susceptibility

To our knowledge, this is the first report of a high-throughput gene expression profiling of MSC from animals genetically selected for their resistance to mastitis. Although, Griesbeck-Hilch *et al. *[31] have already compared the expression of a few genes in mammary epithelial cells (MEC) of mastitis resistant and susceptible cows selected on the basis of the breeding values for SCS or genetic markers. The genetic lines used in this study were created from a one-generation selection of Lacaune dairy ewes based on their parents' breeding values for SCS. The divergence achieved was high, *i.e. *3 genetic standard deviations [22], and was confirmed over successive cohorts raised in the same flock between 2005 and 2009 (RR, unpublished data). The selection criterion used is the SCC, it is based on few repeated measures (usually 3 to 4 times per lactation) and is able to discriminate chronically infected animals from non-infected animals. SCC-based selection, therefore, does not give any information about the underlying mechanisms selected, but previously published results have shown that such selection improves resistance to IMI [22].

In the present study, whereas milk SCC were not significantly different between the lines in the early course of *Staphylococcus *challenges, bacteria titres were lower in the resistant line than in the susceptible line. The difference between lines increased from 12 to 48 hours upon challenge. Accordingly, differentially expressed genes in milk cells at twelve hours post-inoculation might provide useful information about the early mechanisms underlying the genetic control of mastitis in the resistant line, that portend differences of the mastitis issue that were later observed.

#### Cytokines and chemokines

Some soluble factors such as chemokines form gradients from the blood towards infected sites and can enhance neutrophil attraction and migration. In our study, we found no expression differences for major chemokines such as *il-1β*, tumour necrosis factor alpha (*tnfα*)*, il-6 *and *il-8 *(also named *cxcl8*, one of the main chemoattractants for neutrophils [4]), contrary to Griesbeck-Zilch in MEC of mastitis resistant cows selected on QTL alleles [31]. However, integrin beta 6 (*itgb6*), serum amyloid A 2 (*saa2*), a marker of acute inflammation [32,33], S100 calcium binding protein A2 (*s100a2*), a member of the S100 family that is highly correlated with somatic cell count [10], and the chemokine C-C motif ligand 5 (*ccl5*, also named *rantes*) that triggers adhesion and transmigration of blood monocytes to/through endothelial cells were expressed at higher levels in susceptible animals. On the contrary, the expression of calpain 3 (*capn3*) was higher in resistant animals. This protein was shown to play a role in resting neutrophils and to operate as a negative regulator of protrusion and migration [34]. In the present study, although pro-inflammatory molecules have been widely shown to play an important role in neutrophil recruitment and IMI outcome [7,14,28], their expression patterns at 12 hours post-challenge were contrasted in resistant and susceptible lines and not correlated to differences in milk cell concentrations.

#### Neutrophil diapedesis

Furthermore, a group of genes related to cell adhesion and movement, including ST3 beta-galactoside alpha-2,3-sialyltransferase 4 (*st3gal4*), but also activated leukocyte cell adhesion molecule (*alcam*), integrin alpha 5 (*itga5*), selectin platelet (*selp*) and its ligand (*selplg*), were over-expressed in the resistant line. *st3gal4 *is involved in the synthesis of selectin ligand [35]. The selectins are fundamental for the attachment of neutrophils to the endothelial cell surface [4,36] before diapedesis. These results suggest that neutrophil diapedesis might be more efficient in the resistant line.

#### TLR pathways

The genes *cd36 *and *tlr2 *that were expressed at a higher level in resistant animals and that are central in the network analysis collaborate together in the Toll-like receptor (TLR) signalling pathway [37]. TLR2 is dedicated to Gram-positive bacteria recognition [38] and it may enhance the activation of neutrophil phagocytosis [39]. Griesbeck-Hilch *et al. *[31] also found that *tlr2 *was up-regulated in mastitis resistant cows selected on markers for one QTL. The TLR2 signalling pathway may activate a cascade of intracellular events that may initiate the transcription of pro-inflammatory cytokine genes through the ERK/MAPK signalling pathway with consistent up-regulation of *eif4ebp1 *and *itga5 *in the resistant animals. This pathway is involved in oxidative stress which has been shown to contribute to the variability of susceptibility to IMI in cattle [6]. Altogether, the modification of TLR2 and MAPK signalling pathways might be responsible for a higher uptake of bacteria by phagocytes, and therefore might contribute to the more efficient clearance of the infection in the resistant line.

#### Transcription factors

A noteworthy fact is that considerable differences between lines were related to transcriptional activity within MSC. The activator protein 1 (AP-1) transcription factor is considered as an immediate-early response gene and is thought to be involved in a wide range of transcriptional regulatory processes linked to cellular proliferation and differentiation. RARα, TP53, and AHR were also transcription factors of interest.

RARα, the alpha receptor of retinoic acid, a compound derived from vitamin A metabolism, is a nuclear receptor. It can affect several aspects of innate immunity by enhancing the function of neutrophils, macrophages and natural killer cells [40]. It has also been shown to play a role in helping the development of T helper cells, B-cells and, thus antibody-mediated response [40], and more recently in modulating antigen-presenting cells [41]. Retinoic acid metabolism has been shown to play a part in IMI outcome, and dietary vitamin A supplementation has been reported to have a protective effect against experimental *S. aureus *mastitis in mice [42]. Moreover, we found that the aryl hydrocarbon receptor (*ahr*) was under-expressed, which is consistent with several studies that have demonstrated its interaction with RA-signalling pathways [43]. The involvement of AHR in the control of inflammatory responsiveness has been reported previously [44]. To our knowledge the role of TP53 in the response to bacterial infection has yet to be studied whereas it has been shown elsewhere to be associated with mastitis infected quarters [45]. *tp53 *was the gene that displayed the second highest ratio between lines and was over-expressed in the resistant line; it was highly associated with resistant animals in PCA and was represented as a hub in IPA networks since it interacts with numerous other DE genes. *tp53 *can be regulated by S100A2 proteins [46] and JNK [47], and *in vitro *it was shown to regulate TNFα and other cytokines [48]. TP53 has been largely studied in the cancer context for its role in cell cycle arrest, apoptosis, DNA repair and production of antioxidants [49]. Furthermore, TP53 has already been shown to play a pivotal role in determining cellular response to stress via NFκB [50] and TNFα [48]; it may also enhance transcription of the complement regulator CD59 [51]. These observations suggest an important role of TP53 during bacterial infections.

#### Granulopoiesis, cell proliferation, apoptosis

In addition, particular cell functions such as granulopoiesis, cell proliferation and apoptosis seemed to explain some of the differences between the lines. Strong increases in neutrophil efflux from the bone marrow are followed by intense granulopoiesis and efflux of band cells that will later be recruited at the infection site: these cells may show higher transcriptional activity than mature neutrophils. Gene expression analysis demonstrated that the transcription factors CCAAT/enhancer binding protein (C/EBP), alpha (CEBPA) and RARα were up-regulated in the resistant line. CEBPA is crucial for the differentiation of granulocytes [52] and RARα has been shown to be directly involved in some aspects of the immune response by enhancing granulopoiesis [53,54]. Collins *et al. *(60) showed that RARα can enhance granulocytic differentiation through a molecular pathway that is independent of CEBPA [55] suggesting that two different pathways stimulating granulopoiesis were up-regulated in the resistant line.

Furthermore, a number of genes differentially expressed between the sheep lines was related to cell proliferation and apoptosis, e.g. *mapre1, znf259, gzmh cryl1 *and *tp53*. These genes exhibited amongst the highest expression differences between lines. The genes encoding *tp53*, and the lambda-crystallin protein (*cryl1*) were expressed up to five times more in the resistant line. Recently, Cheng *et al. *(61) evidenced association between the expression of CRYL1 and inhibition of cellular proliferation and cell growth [56]. This is in agreement with the decreased expression of both microtubule associated protein RP/EB family member 1 (*mapre1*) and zinc finger protein (*znf259*) in resistant sheep. Indeed, ZNF259 (homologous to ZPR1) has previously been shown to accumulate in the nucleus of proliferating cells [57] and MAPRE1 has been associated during mitosis, with the centrosomes and spindle microtubules. Apoptosis is a critical process necessary to limit or terminate inflammation [6,58] and has previously been shown to be of importance in the response to *S. aureus *by Lutzow *et al. *[10]. The gene expression of *tp53 *has been shown to increase in neutrophils during apoptosis [59]. These results suggest that cell proliferation is limited and apoptosis increased in resistant sheep as compared to susceptible sheep. Altogether data support the hypothesis that, as a consequence of apoptosis/granulopoiesis, the cell turn-over may be enhanced in the resistant line when compared to the susceptible line.

Collectively, these findings highlight leukocyte adhesion and cell migration, pathogen recognition through the TLR2 signalling pathway, and cell turn-over with the balance between apoptosis and granulopoiesis as possible mechanisms to explain a higher susceptibility or resistance to intramammary infection. Nevertheless they probably give only a partial view as other cell types and compartments may be involved (epithelial cells, dendritic cells, lymphocytes, *etc*). Also, other conditions (bacteria strain, time point) might have provided some different results. However, these results advocate the use of our differentially expressed gene list as a benchmark to more detailed genetic studies, including genome co-localisation of resistance to mastitis using QTL analysis as reviewed previously [19,22-25] and polymorphism studies.

## Conclusion

Resistance to mastitis is the consequence of a fine-tuning of immune and inflammatory processes in a complex network of cell and gene interactions. Our study has highlighted some of the possible mechanisms, such as pathogen recognition and neutrophil extravasation leading to improved immune responses against *Staphylococcus *species and consequently, lower susceptibility to infection. The list of the differentially expressed genes between the resistant and susceptible animals provides relevant information for the identification of candidates for the genetic basis underlying resistance to mastitis. It paves the way for further genetic and mechanistic studies.

## Methods

### Animals, experimental challenges and sample collection

To provide enhanced insight into the genetic mechanisms involved in SCS-based selection, two groups of six Lacaune ewes were challenged twice with *Staphylococcus *bacteria. Briefly, primiparous ewes were issued from divergent selection based on extreme breeding values for the somatic cell score (SCS) [22]. On a general basis, the Low SCS ewes are characterised by lower mastitis susceptibility than the High SCS ewes [22]. *Staphylococcus *genus was chosen since it is the most prevalent etiological group in dairy sheep [1] and in SCS lines as previously shown [22]. *Se *and *Sa *bacteria used for inoculation were isolated from ovine chronic mastitis.

After the first lambing, the ewes were inoculated with 10^3 ^cfu of *Se *in a healthy half udder. To clear the infection, local and systemic treatments with antibiotics were applied at the end of the survey. One year later, the same ewes had been mated again, and shortly after the second lambing, they were inoculated with 10^3 ^cfu of *Sa *in the opposite half udder.

Milking was performed by hand twice a day. Milk samples were collected for milk somatic cell count (SCC) every 12-hours from 48 h before to 48 h after the inoculation. SCC was determined using a Fossomatic counter. The score of SCC (SCS) was calculated with the following formula: SCS = 3 + log_2 _(SCC/100,000) [60]. Samples collected aseptically after inoculation were used for bacteriology counts (except the 12 hours post *Sa *inoculation).

Bacteriological analyses were performed at the Veterinary School of Toulouse (UMR 1225 laboratory) by conventional techniques according to International Dairy Federation (1981) guidelines, with a few additions that have already been described in *Rupp et al. *[22]. The bacteriology titre was calculated by a log-10 transformation of the data.

Twelve hours after challenge, mammary glands were emptied and teat ends were disinfected with 70% alcohol. Then, MSC were recovered by cisternal lavage with 100 ml of a saline solution. After centrifugation, cell pellets were resuspended in TRIzol reagent and stored at -80°C until further processed. In parallel, cells were immediately processed for flow cytometry analysis. After incubation with propidium iodide (1 μg/ml final concentration). Data were collected on at least 20,000 events on a FACSCalibur (BD Biosciences) and analysed with FlowJo software.

Statistical analyses of SCS and bacteriological titres were performed with ANOVA applied to mixed models (SAS^® ^v.9.1), and with a non-parametric Wilcoxon test for viability and milk cell population.

Experiments were performed according to French (Agreement number N°31-2010-67) and European rules, and following the regulations of the Animal Ethics Committee for INRA (France).

### Microarray analysis

#### RNA extraction, amplification and labelling

Total RNA was extracted from the 24 cell samples - six ewes from two lines for two challenges - using a typical phenol/chloroform extraction method with Trizol reagent (Invitrogen). Extracted RNA was further purified on Qiagen RNeasy columns (Mini kit, Qiagen). RNA quality was assessed using an Agilent 2100 BioAnalyzer and the RIN (RNA Integrity Number) index was above 7 for all samples.

#### Hybridisation, scanning and raw data storage

For each of the 24 samples, 200 ng of RNA was converted into double-stranded cDNA using the Amino Allyl Message Amp II aRNA amplification procedure (Ambion kit). cDNA was then labelled with Cy3 and Cy5 to obtain 48 dyed samples. Samples were hybridised in a two-colour dye-switch experimental design (GenoToul, France, http://biopuce.insa-toulouse.fr/Maquette/en/) on 22 microarrays of the ovine oligonucleotide 019921 Agilent slide (Agilent Technology).

Chips were hybridised with labelled cDNA at 65°C for 17 hrs and then washed according to the Agilent Technologies protocol. Intensity values were recorded with a 4000B Axon scanner. Two channel images were imported into the Agilent Technology Feature Extraction software for feature spot finding and alignment, and data were normalised with a Loess procedure.

#### Annotation of the ovine microarray

A total of 15,008 different probes are present on the Agilent ovine slide, but only 1,656 genes were annotated by Agilent (version available in January 2010). Moreover, 8,847 genes were identified as Human ortholog Gene Nomenclature Committee (HGNC) by SIGENAE (http://www.sigenae.org/ sheep oligo annotation version 5 of 2009/11/10) [61]. More information about unannotated probes was obtained through the Basic Local Alignment Search Tool programme on the NCBI website (http://blast.ncbi.nlm.nih.gov/Blast.cgi) and the ENSEMBL website (http://www.ensembl.org/index.html). After this annotation phase, only a few focus probes remained unannotated.

#### Processing data and statistical analysis of microarray data

The Feature Extraction result files (.txt) were imported into GeneSpring^® ^GX 11 as single-channel values. Gene expression was analysed probe by probe using an intensity-based model - *i.e. *working on the intensity of spots and not on the ratio between conditions. This way of analysing two-colour-microarray data was shown to enhance the reproducibility of results and the sensitivity of the detection of DE genes [62]. Data were filtered according to spot intensity, saturation and uniformity. Genes were flagged individually for each of the four conditions: low SCS *Sa*, low SCS *Se*, high SCS *Sa *and high SCS *Se*. Only probes that were positively flagged in all samples for at least one condition were conserved for further analysis in order to keep only genes that were very representative of one condition (n = 9,098). Then data were normalised across arrays with the GeneSpring^® ^"scale to median" procedure.

Initially, a two-way ANOVA was performed for each probe to identify DE genes between the two *Staphylococcus *challenges (*Sa *and *Se*) and between the two divergent sheep lines (Low SCS and High SCS). The interaction between the Challenge and the Line effects was tested but was not significant; therefore, it was removed from the statistical model. The p-values of the tests were corrected with a 5% false discovery rate (FDR) with Benjamini-Hochberg [63] and genes with an absolute fold-change (aFC) superior to 1.5 were considered as differentially expressed. For the Line effect, FC represents the ratio between Low and High SCS; for the Challenge effects, it symbolises the ratio between *Sa *and *Se*. The experiment was deposited in GEO at the identifier number GSE24925 (BioArray Software Environment - version SIGENAE).

As 5,573 genes were differentially expressed for the Challenge effect, a second analysis was performed considering only samples infected by one *Staphylococcus *strain to focus on genetic differences between the lines. The whole raw data set was divided into two subsets: MSC from *Sa *and *Se *infections respectively. Data filtering and normalisation were performed as previously for the ANOVA model and 7,452 and 8,561 probes were retained for statistical analysis of the *Se *and *Sa *challenges, respectively. Then, an unpaired Mann Whitney test was performed probe by probe. Considering a 5% FDR no probe was identified as significantly differentially expressed between the lines probably because of the weak number of animals (six in each line). However to further explore the differences of mastitis susceptibility, the p-value threshold of statistics was relaxed. The p-value was rounded to two decimal places, then, the genes with a p-value ≤ 0.01 and an aFC greater than 1.5 were considered as differentially expressed.

Expression profiles for DE genes were classified using the hierarchical clustering algorithm in GeneSpring^® ^based on Pearson-centred gene distances to visualise the differences between the two conditions. They were also represented in principal component analysis (PCA) with the centred data in R (v. 2.9.0) to identify the most important genes to explain mastitis resistance or susceptibility.

#### Biological interpretations of the differentially expressed genes

Three software programmes were used to interpret the lists of focus genes obtained from statistical analysis: Ingenuity Pathway Analysis (IPA), Database for Annotation, Visualization and Integrated Discovery (DAVID v6.7) [64,65] and Innate Data Base (InnateDB) [30].

IPA software (version 7.5, http://www.ingenuity.com/) was used to generate biological networks from a list of selected genes and to document the functions of these genes and the canonical pathways in which they are involved.

Gene Ontology analysis was performed using DAVID (http://david.abcc.ncifcrf.gov/) and led to the establishment of relationships between genes with similar biological functions. Transcription factors that potentially regulate several focus genes were identified with InnateDB (http://www.innatedb.com).

### Reverse transcription

cDNA was generated from 300 ng of clean total RNA from all samples using the Superscript III First Strand Synthesis System Kit (Invitrogen) following the manufacturer's instructions with random hexamer primers and a RNaseH treatment step.

#### Reverse transcription quantitative polymerase chain reaction (RT-qPCR)

The expression of some differentially expressed genes was verified by qPCR. Primer pairs were designed using Primer3 [66] based on the relevant ovine sequences and verified using Primer Express^® ^software. Their specificity was checked with BLAST (http://blast.ncbi.nlm.nih.gov/Blast.cgi). Primers were synthesised commercially by Eurogentec. For genes for which no ovine sequence was available, a comparative gene alignment of bovine, human, rat and mouse sequences was made and primers were then designed on the most conserved regions between the species. Absence of primer dimers was verified using melting curve analysis and the efficiency of the amplification was measured before use. The couples of primers used in qPCR experiments are listed in Additional file 5. qPCR reactions were performed on a 7300 Real-Time PCR System (Applied Biosystems). To validate genes differentially expressed between resistant and susceptible animals, qPCRs were performed either on both *Sa *and *Se *samples or only on *Sa *samples. All assays were carried out in duplicate and each reaction contained 5 μl of diluted cDNA (1:50) with 2.5 μl (0.5 μm) of each forward and reverse primer along with 12.5 μl of Power Syber Green PCR Master Mix (Applied Biosystems).

Specific amplification of each target was confirmed by melting curve analysis. Measured Ct values were exported from SDS software to Excel for data analysis. RT-qPCR technical replicates of samples were averaged. The stability of 7 housekeeping genes, previously cited in the literature, was checked in the 24 samples and data was analysed using GeNorm software [67]. The four most stable genes (*rp19, hprt, sdh *and *gapdh) *were selected for normalisation of RT-qPCR. Fold changes were calculated by the delta delta Ct method normalised to the four housekeeping genes [67] with R (version 2.9.0). Statistical analysis was performed using an exact non parametric Wilcoxon test with SAS (version 9.1).

## Authors' contributions

CB carried out the microarray experiment, performed the statistical analysis and drafted the manuscript. GF and RR designed the experiment, supervised the analysis and participated in writing the paper. RR designed the divergent selection experiment. MRA bred the animals. GF supervised experimental challenges and sample collection. DB, EF, CT and CC participated in the experimental challenges. DB supervised the bacteriology analyses. EF and SB performed flow cytometry analysis. CC prepared RNA for hybridisation. CC, MT and CB hybridised the samples. MT was in charge of the RT-qPCR analysis. RR, CRG and GF helped and discussed the statistical methods implemented. All authors read, helped to edit and approved the final manuscript.

## Supplementary Material

Additional file 1 **List of the differentially-expressed genes between *S. aureus *and *S. epidermidis *challenges**. Analysis was performed with GeneSpring^®^. The enumerator of the FC represents the *Sa *samples and the denominator the *Se *samples. Genes were considered as differentially expressed if the FDR q-value < 0.05 and the absolute FC > 5 (n = 261 probes, n = 210 genes). Click here for file

Additional file 2 **Networks of the differentially expressed genes between *S. aureus *and *S. epidermidis *challenges**. Network analysis was performed with IPA. Genes up-regulated after *Sa *challenge are in red whereas genes up-regulated after *Se *challenge are in green. (A). The main biological functions of the network A (molecules: 26, score: 42) are cell-to-cell signalling and interaction, cell-mediated immune response. (B). The main biological functions of the second network (molecules: 15, score 21) are inflammatory disease, inflammatory response and haematological disease. Click here for file

Additional file 3 **List of the differentially expressed genes between the resistant and susceptible lines**. Analysis was performed with GeneSpring^® ^(n = 380 probes, n = 335 genes). The fold change and the p-values of the three analyses are present. ANOVA stands for analysis with all data (corrected p-value with a FDR of 5% and absolute FC > 1.5); *Sa*, for analysis with only *S. aureus *data, and *Se *for analysis with only *S. epidermidis *data (p-value ≤ 0.01 and FC > 1.5). Click here for file

Additional file 4 **Biological process GO Terms of the differentially expressed genes between the resistant and susceptible lines**. Analysis was performed by DAVID (n = 335 genes, n = 306 genes recognised by DAVID). Of the 160 and 146 up- and down-regulated genes, 126 and 119 GO terms were identified, respectively. "Count" stands for the number of differentially expressed genes in a GO Term class. "%" represents the number of genes involved in given term divided by the total number of input genes, *i.e. *percentage of input genes hitting a given term. "Hit" is the number of TF binding sites and the "Enrichment Fold" measures the magnitude of enrichment. Click here for file

Additional file 5 **Oligonucleotide sequences for quantitative PCR**. The sequences of the couples of primers to confirm the Challenge effect are listed in the table A and the ones for the Line effect in the table B. Click here for file
