# Supplementary figures and images for: Transcriptomic analysis of milk somatic cells in mastitis resistant and susceptible sheep upon challenge with Staphylococcus epidermidis and Staphylococcus aureus
Source: BMC Genomics. 2011 Apr 28;12:208. doi: 10.1186/1471-2164-12-208 (PMC3096985; doi:10.1186/1471-2164-12-208)

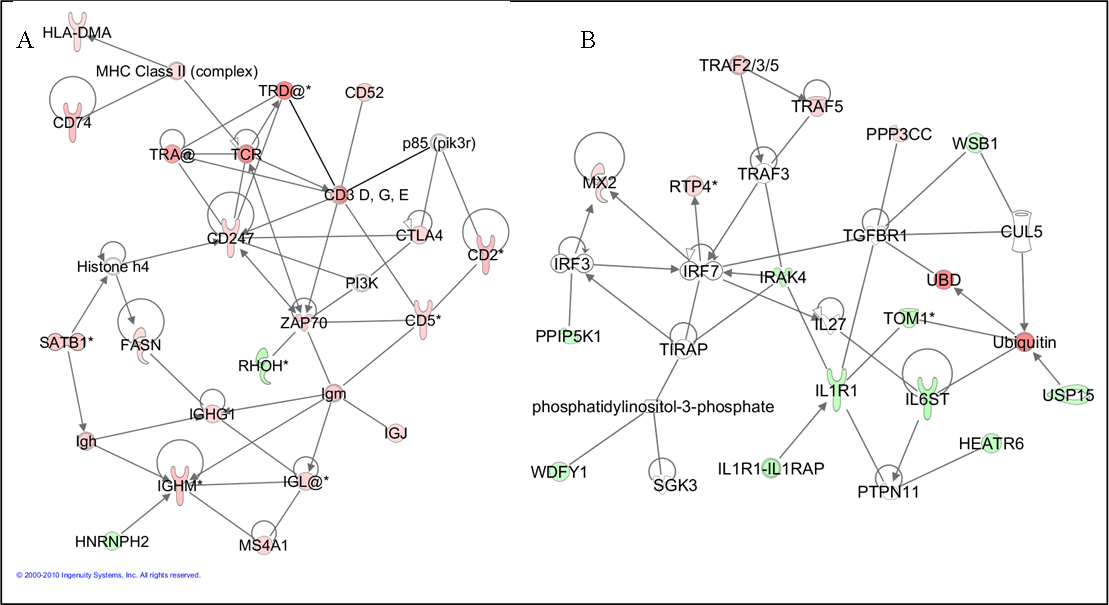

Supplement: Additional file 2 — Networks of the differentially expressed genes between S. aureus and S. epidermidis challenges. Network analysis was performed with IPA. Genes up-regulated after Sa challenge are in red whereas genes up-regulated after Se challenge are in green. (A). The main biological functions of the network A (molecules: 26, score: 42) are cell-to-cell signalling and interaction, cell-mediated immune response. (B). The main biological functions of the second network (molecules: 15, score 21) are inflammatory disease, inflammatory response and haematological disease. [file 1471-2164-12-208-S2.PNG]
